# A Survey on Scabies Inpatients in South Korea Based on Health Insurance Claims Data from 2010 to 2019

**DOI:** 10.3390/healthcare11060841

**Published:** 2023-03-13

**Authors:** Hyung-Seon Kim, Jji-Ya Bang, Kyung-Sook Cha

**Affiliations:** 1 Department of Nursing, Bucheon University, 56 Sosa-ro, Bucheon-si 14774, Gyeonggi-do, Republic of Korea; 2 Medical Supply Benefits Listing Division, Health Insurance Review & Assessment Service, 60 Hyeoksin-ro, Wonju-si 26465, Gangwon-do, Republic of Korea; 3 Department of Nursing Science, Sun Moon University, 70 Sunmoon-ro 221 beon-gil, Tangjeong-myeon, Asan-si 31460, Chungcheongnam-do, Republic of Korea

**Keywords:** scabies, inpatients, medical, utilization

## Abstract

Due to the growing aging population and the increased number of long-term patients staying in nursing facilities, the prevalence of scabies has recently been increasing, even in developed countries. This study aimed to identify the actual status of hospitalized patients with scabies in South Korea using the national health insurance claims data. From 2010 to 2019, 2586 patients were hospitalized with scabies (B86) as the primary diagnosis. There were more females than males (χ^2^ = 31.960, *p* < 0.001) and patients aged 80 years or older in long-term care hospitals (χ^2^ = 431.410, *p* < 0.001). Scabies patients were mainly hospitalized in internal medicine, family medicine, and dermatology for all provider types (χ^2^ = 170.033, *p* < 0.001). In long-term care hospitals, the rate of accompanying dementia was 31.9% (χ^2^ = 193.418, *p* < 0.001), cerebral infarction was 10.4% (χ^2^ = 106.271, *p* < 0.001), and cancer was 2.1% (χ^2^ = 17.963, *p* < 0.001), which was higher than other provider types. Additionally, 20.6% in general hospitals (χ^2^ = 198.952, *p* < 0.001) had an indwelling catheter, while 49.1% in hospitals and 41.1% in general hospitals were administered steroids (χ^2^ = 214.440, *p* < 0.001). The KOH smear test was performed in 11.3% of all inpatients with scabies. We suggest recognizing these characteristics of scabies patients and thoroughly checking the skin lesions during physical examination for early diagnosis and prevention of scabies infection.

## 1. Introduction

Scabies is an infectious skin disease caused by *Sarcoptes scabiei var. hominis* and is classified as general and crusted scabies (Norwegian scabies). The symptoms of scabies vary according to the severity of the scabies, the number of mites, and the time from initial infection to diagnosis [1]. The most common symptom is severe itching due to skin lesions, which causes patients to scratch the lesions, resulting in secondary bacterial infections. In addition, it has been reported that there is a high risk of glomerulonephritis [2], chronic kidney disease [3], and stroke [4] after a major infection. Scabies mite infection spreads rapidly through direct skin contact, not only via patients but also via asymptomatic carriers [5]. Scabies seriously impairs patients’ quality of life due to itching and sleep disturbance while causing significant financial losses to medical institutions due to continuous infection control costs and a negative reputation for scabies outbreaks [6].

Scabies is globally distributed and is estimated to affect up to 300 million people worldwide [7], covering all ages and social classes. Industrialization, economic poverty, poor sanitation, population overcrowding, and malnutrition have been reported as risk factors for infection. High incidence rates have been reported in tropical regions and developing countries where there are many risk factors for infection [8,9]. However, due to the growing aging population and the increased number of long-term patients staying in nursing facilities, the prevalence of scabies infection has recently been increasing, even in developed countries.

In France, the number of patients treated for scabies increased from 1999 to 2010, and the prevalence of scabies was reported to be 328 per 100,000 population [10]. In developed countries, the main cause of the increase in the incidence of scabies is presumed to be the failure of scabies treatment [11].

Scabies is regularly identified in patients in South Korea. The number of patients with scabies who are 50 years or older in South Korea has been increasing, and the number of medical benefit patients who were billed to the Health Insurance Review and Assessment Service (HIRA) with a scabies diagnosis code (B86) fluctuated from 51,331 in 2010 to 36,579 in 2020. In particular, over the past ten years, there was a 3% average annual increase in scabies in males over 80, while the incidence of scabies in females aged 60–69 increased by 8% [12]. There has been a continuous increase in the number of females with scabies compared to males, and, on average, the incidence of scabies is lowest in spring and highest in autumn [13].

Given the prevalence, contagiousness, and consequences of scabies, measures to prevent its spread are essential. Nevertheless, many regions do not recognize scabies as a significant public health issue [14]. Although the number of patients with scabies continues to increase in South Korea, there is a lack of studies that identify the national situation of patients with scabies or the risk factors for outbreaks.

Therefore, this study aimed to investigate the status of hospitalized patients with scabies in South Korea by utilizing nationwide health insurance claims data from the HIRA.

## 2. Materials and Methods

### 2.1. Study Design

This descriptive study was conducted to investigate the actual conditions of patients with scabies admitted to tertiary hospitals, general hospitals, hospitals, and long-term care hospitals.

### 2.2. Subjects

The subjects of this study were patients who were hospitalized and treated for scabies (B86) in tertiary hospitals, general hospitals, hospitals, and long-term care hospitals from 2010 to 2019. In total, 2586 of inpatients with scabies as their primary diagnosis (B86) were included in this study.

### 2.3. Definition of Variables

#### 2.3.1. Number of Inpatients with Scabies

In this study, the inpatients with scabies were those inpatients who had claimed medical care benefits for scabies (B86).

#### 2.3.2. Provider Type

The types of providers were tertiary hospitals, general hospitals, hospitals, and long-term care hospitals.

#### 2.3.3. General Characteristics of the Inpatients with Scabies

The general characteristics of the inpatients with scabies were classified by sex, insurance type (health insurance, medical aid), age, medical department, and location of medical institutions.

#### 2.3.4. Treatment Characteristics of the Inpatients with Scabies

The treatment characteristics of the inpatients with scabies were classified according to the presence of comorbidities, whether indwelling catheters were inserted, whether steroids were administered, and whether a microscopic examination was performed. Comorbidities included dementia, hypertension, diabetes, cerebral infarction, end-stage renal disease (ESRD), chronic obstructive pulmonary disease (COPD), and cancer. The drugs included in steroid preparations include crotamiton, lindane, benzyl benzoate, permethrin, and ivermectin.

### 2.4. Ethical Considerations and Data Collection

This study was approved by the Institutional Review Board (SM-202008-055-1). The researcher received approval for data use through deliberation by the Data Provision Deliberation Committee of the HIRA. After receiving approval, the data were analyzed remotely using the Healthcare Bigdata Hub system in the HIRA. The data used in the analysis were the hospitalization claims data requested by the HIRA for patients with scabies with health insurance and medical aid admitted to tertiary hospitals, general hospitals, hospitals, and long-term care hospitals from 2010 to 2019.

### 2.5. Data Analysis

The data were analyzed using SPSS/WIN version 25.0. The status of the inpatients with scabies by provider type was calculated by frequency and percentage, and the general and treatment characteristics of the inpatients with scabies were analyzed using the chi-square test (χ^2^ test). The statistical significance level for the data was set at *p* < 0.05.

## 3. Results

### 3.1. Status of the Inpatients with Scabies by Provider Type

The status of the inpatients with scabies according to provider type is shown in Table 1. From 2010 to 2019, 2586 patients were hospitalized with scabies (B86) as the primary diagnosis: 268 in tertiary hospitals (10.4%), 879 in general hospitals (34.2%), 989 in hospitals (38.5%), and 432 in long-term care hospitals (16.8%). The medical institutions with the highest average annual growth rate of scabies were long-term care hospitals, which increased by 14.7%, whereas hospitals decreased by 9.5%.

### 3.2. General Characteristics of the Inpatients with Scabies by Provider Type

The general characteristics of the inpatients with scabies according to provider type are shown in Table 2. As for the sex of the inpatients with scabies, there were more males than females in tertiary hospitals, general hospitals, and hospitals, while there were more females than males in long-term care hospitals, which was statistically significant (χ^2^ = 31.960, *p* < 0.001). In terms of insurance, there were more patients with health insurance in tertiary hospitals, general hospitals, and long-term care hospitals, and more medical aid patients in hospitals, which was statistically significant (χ^2^ = 422.044, *p* < 0.001). Scabies was most commonly diagnosed in patients aged 60–79 years in tertiary and general hospitals, patients aged 40–59 years in hospitals, and patients aged 80 years or older in long-term care hospitals, which was statistically significant (χ^2^ = 431.410, *p* < 0.001). The medical institutions that had inpatients with scabies were mostly local for all provider types, which was statistically significant (χ^2^ = 135.017, *p* < 0.001).

### 3.3. Treatment Characteristics of the Inpatients with Scabies by Provider Type

The treatment characteristics of the inpatients with scabies according to provider type are shown in Table 3. The medical departments with the most inpatients with scabies were internal medicine, family medicine, dermatology, general surgery, and urology in all provider types, which was statistically significant (χ^2^ = 170.033, *p* < 0.001). The prevalence of comorbidities was assessed among inpatients with scabies, and the results showed that 12.3% of patients had comorbid dementia, which increased to 31.9% in long-term care hospitals (χ^2^ = 193.418, *p* < 0.001). The prevalence of comorbid hypertension was 24.6% in tertiary hospitals and 24.3% in long-term care hospitals (χ^2^ = 66.029, *p* < 0.001). Furthermore, 23.9% of inpatients with scabies in tertiary hospitals (χ^2^ = 28.906, *p* < 0.001) and 10.4% in long-term care hospitals (χ^2^ = 106.271, *p* < 0.001) had diabetes. Moreover, 3.7% (χ^2^ = 22.354, *p* < 0.001) and 3.0% (χ^2^ = 13.251, *p* = 0.004) of inpatients with scabies in tertiary hospitals had ESRD and COPD, respectively. The incidence of cancer was 2.1% in long-term care hospitals (χ^2^ = 17.963, *p* < 0.001). Furthermore, 20.6% of cases in general hospitals (χ^2^ = 198.952, *p* < 0.001) had an indwelling catheter inserted, and 49.1% in hospitals and 41.1% in general hospitals were administered steroids, respectively (χ^2^ = 214.440, *p* < 0.001). KOH smear tests were performed in 27.5% and 22.8% of patients in long-term care and tertiary hospitals, respectively (χ^2^ = 241.839, *p* < 0.001).

## 4. Discussion

This study aimed to investigate the status of hospitalized patients who claimed medical care benefits to HIRA for scabies (B86) as their primary diagnosis in South Korea from 2010 to 2019.

Scabies spread across South Korea in the 1970s, particularly among teenagers and people in their 20s and 30s. The incidence rate has decreased rapidly with economic growth, and scabies now occurs most often in people over 60 years old [8]. As confirmed in this study, the incidence of patients aged ≥60 years accounted for 61.1% of the total, and the number of patients admitted to long-term care hospitals, which was about 10–20 anually from 2010 to 2013, gradually increased to 106 in 2019. This is related to an increase in the number of nursing facilities for the elderly. The number of nursing facilities for the elderly has increased significantly, from approximately 1300 in 2008 to about 4000 in 2021 [15]. Because the probability of infection of a disseminated disease through contact is increased in these facilities due to the patients living in close proximity [16], it has been suggested that the occurrence and management of infectious diseases in long-term care facilities should be considered a new public health problem.

Scabies is a disease that can be transmitted through direct contact with an infected person, regardless of personal hygiene. In Japan, it was reported that 41% of psychiatric and long-term care hospitals experienced scabies in 2004 [17]. In Canada, 25% of 130 chronic healthcare institutions reported experiencing scabies in one year [18]. In Korea, 22.7% of 798 long-term care hospitals reported the occurrence of scabies in one year [19], confirming that scabies occurs frequently in long-term care hospitals. However, the occurrence of scabies is not only a problem in long-term care hospitals. As a result of this study, the number of scabies inpatients in tertiary and general hospitals showed a continuous increase. Because the Korean healthcare delivery system allows patients to move freely between medical institutions, if the condition of a patient in a long-term care hospital deteriorates or an acute phase problem occurs, the patient is transferred to a tertiary hospital or general hospital and can be transferred back to a long-term care hospital after acute treatment is completed [20]. However, while patients can move freely between institutions, the sharing of patient information is limited. The institution receiving the transfer cannot view the patient’s medical information in the previous institution and can only check the information provided by the institution. Therefore, if sufficient information is not shared, patient detection may be delayed, leading to an epidemic of infection in medical institutions. Therefore, it can be seen that the increase in scabies in long-term care hospitals is related to the increase in prevalence in higher institutions.

The hospitals that had inpatients with scabies were mostly local for all provider types. Scabies infection is affected by economic level, sanitary conditions, immunity, and nutritional deficiencies [8,9]. In Korea, the economic level of rural areas is relatively low compared with cities [21], and the elderly population is high [22]. Considering that there may be problems with health and hygiene management due to the high proportion of elderly households whose owners are aged 65 years or older [22], it is estimated that many inpatients with scabies are distributed in local medical institutions.

In this study, the most frequent comorbidities among the inpatients with scabies were diabetes (16.6%), hypertension (16.4%), dementia (12.3%), cerebral infarction (2.9%), ESRD (1.4%), and COPD (1.4%). In a study by Wang et al. (2012), which identified the risk factors for scabies, diabetes accounted for more than 50% of the underlying diseases in both patients with and without scabies. In a study by Lee et al. [23], there was no significant difference in the occurrence of scabies according to the presence or absence of diabetes and hypertension, which are underlying diseases.

Therefore, hypertension and diabetes, which are frequent comorbidities, are not considered risk factors for scabies. Considering that more than 50% of the inpatients with scabies were over 60 years of age in the current study, we assumed that these two comorbidities are more likely to occur in older people.

In long-term care hospitals, the prevalence rates of scabies accompanying dementia, cerebral infarction, and hypertension were relatively higher than those in higher institutions. In a study [24] that identified the prevalence of chronic diseases in older adults admitted to nursing facilities and long-term care hospitals, the prevalence rates of hypertension (40.2% and 54.9%, respectively) and dementia (38.6% and 55.4%, respectively) were the highest, while the rates of stroke patients were 19.5% and 25.7%, respectively. These results are in line with those of this study. Wang et al. [25] reported that the scabies patient group had a higher medical history of ischemic or cerebrovascular accidents than the control group and that the rate of being unable to speak or being in a bedridden state was higher. In the case of patients with dementia or stroke, it may be difficult to express symptoms such as itchiness due to difficulties in self-expression, and there may be limitations in maintaining personal hygiene due to activity restrictions. In addition, Makigami et al. [26] reported that the incidence and recurrence rates of scabies are high in patients with dementia. They stated that in the case of patients with dementia, in addition to frequent exposure to infectious agents, problematic behaviors such as lying in bed with other patients can increase the recurrence rate of scabies. Therefore, it is necessary to strictly prevent contact with patients and thoroughly manage the environment to prevent the occurrence of scabies.

The number of patients who underwent indwelling catheter insertion was higher in tertiary hospitals (17.5%), general hospitals (20.6%), hospitals (3.5%), and long-term care hospitals (1.4%). Patients with indwelling catheter insertion are more likely to be immobilized or severely ill, which could explain why the insertion rate was higher in patients admitted to tertiary and general hospitals than in patients admitted to long-term care hospitals. In some studies, the risk factors for scabies have been confirmed to include the use of catheters (nasogastric tube, Foley catheter, Port-A, and Hickman catheter) [25] or clinically severe cases at hospitalization [23,25]. Patients with severe medical conditions require intensive acute treatment, and in this situation, the medical staff has limited interest in diagnosing scabies [23]. In addition, when a catheter such as an indwelling catheter is inserted and maintained, it may affect the occurrence of scabies due to difficulties in performing personal hygiene tasks, such as bathing.

If scabies is confirmed, it is first treated with anti-scabies mites; if dermatitis occurs due to an allergic reaction caused by the excretion of scabies mites, steroids are also used [8]. In this study, 37.5% of the patients were administered steroids during hospitalization, and the rate of administration in hospitals (49.1%) and general hospitals (41.1%) was higher than that in long-term care hospitals (9.7%). It seems that the reason for such a low dosage rate in long-term care hospitals is that a comprehensive amount per day for hospitalization is applied to them, except for diseases to which a fee-for-service is applied, such as pneumonia and sepsis. This suggests the possibility that long-term care hospitals do not carry out active administrations compared with medical institutions that are subject to the fee-for-service system.

In this study, the KOH smear test was performed in 11.3% of all inpatients with scabies, confirming that scabies was diagnosed and treated in many cases without skin examination. Scabies can be diagnosed by characteristic nighttime itching, the discovery of scabies mite burrows, and a history of exposure, in addition to a KOH smear test that identifies mites, eggs, or excreta in the papules or burrows [8,27]. In other words, in addition to the confirmation test, it is also possible to conduct a multilateral evaluation, such as checking for physical symptoms and exposure history for diagnosis. However, in patients with neuropathy or cognitive impairment, a diagnosis can be challenging as these conditions can change the patient’s behavior and/or their ability to express their symptoms [28]. In particular, scabies lesions can appear in the inguinal and axillary areas; therefore, it is necessary to conduct a thorough physical examination because such lesions can be missed if the physical examination is performed without the patient’s clothes off. Many scabies-infected individuals are asymptomatic [29], and non-specific dermatitis is common, especially in the elderly. In addition, scabies is similar to other skin diseases, such as atopic dermatitis or psoriasis, requiring differential diagnosis in many cases [9]. These factors can increase the infectivity of scabies. Therefore, if someone is considered at high risk for scabies infection and shows suspicious symptoms or a history of exposure, a preemptive confirmatory test is required.

There is a high risk of infectious diseases spreading in medical institutions due to the high number of patients who are vulnerable to infection being gathered in a confined space. Scabies is not associated with a high mortality rate; however, it can spread between patients or medical staff through contact. In particular, crusted scabies is highly likely to spread quickly due to its excellent dissemination power. Infection control in patients with scabies is important for preventing and managing healthcare-associated infections, and in the case of elderly patients, special attention is needed because the early diagnosis and treatment of scabies can prevent secondary infections and sequelae. Therefore, to promptly diagnose patients with scabies, medical staff must be familiar with the various aspects of scabies and conduct a thorough physical examination. In addition, appropriate education should be provided to healthcare workers caring for patients at high risk of scabies.

## 5. Conclusions

This study analyzed the data of inpatients who claimed medical care benefits for scabies as their primary diagnosis. While these data do not indicate the actual incidence rate, they are significant because they provide basic data by confirming the incidence trend and characteristics of inpatients with scabies by provider type.

From 2010 to 2019, patients with scabies who claimed medical care benefits showed an annual increase of 1.7%, and in 2019, a rapid increase in the number of inpatients in long-term care hospitals was confirmed. As a result of confirming the characteristics of the inpatients with scabies, the proportion of patients over 60 years of age with dementia as an underlying disease and who had an indwelling catheter inserted was high. This study is an investigation of the characteristics of patients hospitalized with scabies, and not a study that confirms the causal relationship between these characteristics and scabies. Therefore, interpretation should be carried out with caution. However, if healthcare workers recognize the previously mentioned characteristics, it could help promote the early diagnosis and infection prevention of scabies and help promote thorough checks of the presence or absence of skin lesions during physical examinations.

Scabies is not legally designated as a notifiable disease in South Korea, making it challenging to ascertain the actual incidence of scabies. Additionally, the limited number of cases originating from a single institution presents a challenge in accurately identifying risk factors. Therefore, we propose a multi-institutional study over several years to identify nationwide incidence trends and risk factors for scabies. Based on these findings, we recommend developing guidelines that aid in the early detection and prevention of scabies infection in high-risk patients.

## Figures and Tables

**Table 1:** Total Number of Scabies Inpatients in Tertiary Hospitals, General Hospitals, Hospitals, and Long-Term Care Hospitals (2010–2019).

Categories	Year (%)	2010	2011	2012	2013	2014	2015	2016	2017	2018	2019	Average Annual Increase Rate
Total	2568 (100.0)	263	242	260	230	208	213	253	321	267	311	1.7%
Tertiary hospitals	268 (10.4)	17	31	21	19	41	22	31	34	23	29	5.5%
General hospitals	879 (34.2)	61	97	75	84	60	74	93	98	119	118	6.8%
Hospitals	989 (38.5)	158	93	153	117	77	80	54	124	75	58	−9.5%
Long-term care hospitals	432 (16.8)	27	21	11	10	30	37	75	65	50	106	14.7%

**Table 2:** General Characteristics of Scabies Inpatients by Provider Type.

Variables	Categories	Total	Tertiary Hospitals	General Hospitals	Hospitals	Long-Term Care Hospitals	χ^2^ (*p*)
		n (%)	n (%)	n (%)	n (%)	n (%)	
Total score		2586 (100.0)	268 (100.0)	879 (100.0)	989 (100.0)	432 (100.0)	
Gender	Male	1387 (54.0)	149 (55.6)	462 (52.6)	588 (59.5)	188 (43.5)	31.960
	Female	1181 (46.0)	119 (44.4)	417 (47.4)	401 (40.5)	244 (56.5)	(<0.001)
Medical Benefits	Health Insurance	1375 (53.5)	191 (71.3)	592 (67.3)	278 (28.1)	314 (72.7)	422.044
	Medical Aid	1193 (46.5)	77 (28.7)	287 (32.7)	711 (71.9)	118 (27.3)	(<0.001)
Age	0–19	121 (4.7)	17 (6.3)	50 (5.7)	54 (5.5)	0 (0.0)	431.410
	20–39	180 (7.0)	13 (4.9)	44 (5.0)	117 (11.8)	6 (1.4)	(<0.001)
	40–59	697 (27.1)	62 (23.1)	161 (18.3)	435 (44.0)	39 (9.0)	
	60–79	830 (32.3)	94 (35.1)	327 (37.2)	227 (23.0)	182 (42.1)	
	≥80	740 (28.8)	82 (30.6)	297 (33.8)	156 (15.8)	205 (47.5)	
Location	Seoul	301 (11.7)	63 (23.5)	126 (14.3)	64 (6.5)	48 (11.1)	135.017
	Metropolitan	540 (21.0)	72 (26.9)	189 (21.5)	149 (15.1)	130 (30.1)	(<0.001)
	Province	1727 (67.3)	133 (49.6)	564 (64.2)	776 (78.5)	254 (58.8)	

**Table 3:** Medical Treatment Characteristics of Scabies Inpatients by Provider Type.

Characteristics	Categories	Total	Tertiary Hospitals	General Hospitals	Hospitals	Long-Term Care Hospitals	χ^2^ (*p*)
		n (%)	n (%)	n (%)	n (%)	n (%)	
Total score		2568 (100.0)	268 (100.0)	879 (100.0)	989 (100.0)	432 (100.0)	
Medical Department	Internal Medicine	739 (28.8)	84 (31.3)	239 (27.2)	308 (31.1)	108 (25.0)	170.033
	Family Medicine	542 (21.1)	38 (14.2)	140 (15.9)	271 (27.4)	93 (21.5)	(<0.001)
	Dermatology	332 (12.9)	50 (18.7)	129 (14.7)	91 (9.2)	62 (14.4)	
	General Surgery	304 (11.8)	31 (11.6)	114 (13.0)	113 (11.4)	46 (10.6)	
	Urology	221 (8.6)	24 (9.0)	91 (10.4)	75 (7.6)	31 (7.2)	
	Rehabilitation Medicine	79 (3.1)	6 (2.2)	42 (4.8)	11 (1.1)	20 (4.6)	
	Emergency Medicine	76 (3.0)	9 (3.4)	19 (2.2)	22 (2.2)	26 (6.0)	
	Neurology	73 (2.8)	2 (0.7)	28 (3.2)	31 (3.1)	12 (2.8)	
	Neurosurgery	54 (2.1)	5 (1.9)	24 (2.7)	20 (2.0)	5 (1.2)	
	Orthopedic Surgery	45 (1.8)	4 (1.5)	21 (2.4)	10 (1.0)	10 (2.3)	
	Pediatrics	35 (1.4)	3 (1.1)	10 (1.1)	12 (1.2)	10 (2.3)	
	Psychiatry	28 (1.1)	3 (1.1)	6 (0.7)	16 (1.6)	3 (0.7)	
	Etc.	40 (1.6)	9.0 (3.4)	16.0 (1.8)	9.0 (0.9)	6.0 (1.4)	
Dementia	Yes	315 (12.3)	29 (10.8)	85 (9.7)	63 (6.4)	138 (31.9)	193.418 (<0.001)
	No	2253 (87.7)	239 (89.2)	794 (90.3)	926 (93.6)	294 (68.1)	
Hypertension	Yes	420 (16.4)	66 (24.6)	153 (17.4)	96 (9.7)	105 (24.3)	66.029 (<0.001)
	No	2148 (83.6)	202 (75.4)	726 (82.6)	893 (90.3)	327 (75.7)	
Diabetes Mellitus	Yes	427 (16.6)	64 (23.9)	174 (19.8)	125 (12.6)	64 (14.8)	28.906 (<0.001)
	No	2141 (83.4)	204 (76.1)	705 (80.2)	864 (87.4)	368 (85.2)	
Cerebral Infarction	Yes	74 (2.9)	6 (2.2)	10 (1.1)	13 (1.3)	45 (10.4)	106.271 (<0.001)
	No	2494 (97.1)	262 (97.8)	869 (98.9)	976 (98.7)	387 (89.6)	
ESRD ^†^	Yes	35 (1.4)	10 (3.7)	12 (1.4)	3 (0.3)	10 (2.3)	22.354 (<0.001)
	No	2533 (98.6)	258 (96.3)	867 (98.6)	986 (99.7)	422 (97.7)	
COPD ^‡^	Yes	36 (1.4)	8 (3.0)	13 (1.5)	5 (0.5)	10 (2.3)	13.251 (0.004)
	No	2532 (98.6)	260 (97.0)	866 (98.5)	984 (99.5)	422 (97.7)	
Cancer	Yes	20 (0.8)	2 (0.7)	9 (1.0)	0 (0.0)	9 (2.1)	17.963 (<0.001)
	No	2548 (99.2)	266 (99.3)	870 (99.0)	989 (100.0)	423 (97.9)	
Foley Catheter	Yes	269 (10.5)	47 (17.5)	181 (20.6)	35 (3.5)	6 (1.4)	198.952 (<0.001)
	No	2299 (89.5)	221 (82.5)	698 (79.4)	954 (96.5)	426 (98.6)	
Steroid Therapy	Yes	964 (37.5)	75 (28.0)	361 (41.1)	486 (49.1)	42 (9.7)	214.440 (<0.001)
	No	1604 (62.5)	193 (72.0)	518 (58.9)	503 (50.9)	390 (90.3)	
KOH smear	Yes	290 (11.3)	61 (22.8)	94 (10.7)	16 (1.6)	119 (27.5)	241.839 (<0.001)
	No	2278 (88.7)	207 (77.2)	785 (89.3)	973 (98.4)	313 (72.5)	

^†^ ESRD (end-stage renal disease), ^‡^ COPD (chronic obstructive pulmonary disease).

## Data Availability

Restrictions apply to the availability of these data. The data used and/or analyzed in this study can be used after obtaining approval from the Health Insurance Review and Assessment Service.
